# Using community theater to improve demand for vaccination services in the Niger Delta Region of Nigeria

**DOI:** 10.1186/s12919-023-00263-0

**Published:** 2023-07-03

**Authors:** Chijioke Chikere Kaduru, Geraldine Chinonso Mbagwu, Dumale Koko Aadum, Ganiyat Eshikhena, Godwin Anusa Idim, Uche Francis Ibe, Timiebiere Sabenus, Fofah Gawain Jenson, Edmund Egbe, Neni Aworabhi-Oki, Happiness Masa, Martins Bekesu, Abisoye Sunday Oyeyemi

**Affiliations:** 1 Corona Management Systems: 2B Samuel A. Ogedengbe Crescent Jabi, Abuja FCT, Nigeria; 2 WHO – World Health Organization: WHO office, State Secretariat Complex, Third Floor, Yenagoa, Bayelsa State Nigeria; 3 Bayelsa State Primary Healthcare Board: State Secretariat Complex Third Floor, Yenagoa, Bayelsa State Nigeria; 4 Department of Community Medicine, Niger Delta, University, Wilberforce Island, Bayelsa State Nigeria

**Keywords:** Immunization, Human-centered design, Community theater, Storytelling, Niger Delta, Play

## Abstract

**Introduction:**

Despite abundant evidence showing immunization as a lifesaving public health measure, a large proportion of Nigerian children are still not or fully vaccinated. Lack of awareness and distrust of the immunization process by caregivers are some of the reasons for poor immunization coverage which need to be addressed. This study aimed at improving vaccination demand, acceptance and uptake in Bayelsa and Rivers State, both in the Niger Delta Region (NDR) of Nigeria through a human-centered process of trust building, education and social support.

**Methods:**

A quasi-experimental intervention christened Community Theater for Immunization (CT4I) was deployed in 18 selected communities between November 2019 and May 2021 in the two states. In the intervention localities, relevant stakeholders including the leadership of the health system, community leaders, health workers and community members were engaged and actively involved in the design and performance of the theaters. The content for the theater showcased real stories, using a human-centered design (HCD) of ideation, co-creation, rapid prototyping, feedback collection and iteration. Pre- and post-intervention data on the demand and utilization of vaccination services were collected using a mixed method.

**Results:**

In the two states, 56 immunization managers and 59 traditional and religious leaders were engaged. Four broad themes implicating user and provider factors emerged from the 18 focus group discussions as responsible for low immunization uptake in the communities. Of the 217 caregivers trained on routine immunization and theater performances, 72% demonstrated a knowledge increase at the post-test. A total of 29 performances attended by 2,258 women were staged with 84.2% of the attendees feeling satisfied. At the performances, 270 children received vaccine shots (23% were zero-dose). There was a 38% increase in the proportion of fully immunized children in the communities and 9% decline in the proportion of zero-dose children from baseline.

**Conclusion:**

Both demand- and supply-side factors were identified as responsible for poor vaccination in the intervention communities. Our intervention demonstrates that caregivers will demand immunization services if they are engaged through community theater using a human-centered design (HCD). We recommend a scaling up of HCD to address the challenge of vaccine hesitancy.

**Supplementary Information:**

The online version contains supplementary material available at 10.1186/s12919-023-00263-0.

## Introduction

Immunization continues to be a highly efficient strategy in preventing and managing severe infectious diseases, offering significant cost benefits. An extensive study assessing the health and economic outcomes of routine immunization (RI) from 2001 to 2020 revealed compelling results. It projected that the implementation and expanded coverage of vaccines would prevent more than 14 million deaths, reduce over 350 million illness cases, avert 8 million instances of long-term disability, and save approximately 700 million disability-adjusted life years [1]. Immunization plays a crucial role in achieving the Sustainable Development Goals (SDGs). Specifically, Target 3 of the SDGs focuses on decreasing mortality rates among children under the age of 5 [2].

While vaccination has achieved remarkable success in reducing the impact of childhood diseases and improving child survival rates on a global scale, there still exists inadequate immunization coverage in numerous regions, especially in low- and middle-income countries (LMICs) [3]. The reasons behind the inadequate coverage can be categorized into two main factors: supply-side and demand-side. The supply-side factors involve issues related to access and failures within the healthcare system that result in irregular vaccine availability. Disruptions in vaccination programs and services can occur due to factors such as industrial action by healthcare workers, conflicts, and natural disasters. On the other hand, demand-side factors encompass psychological and socio-cultural reasons. These include misconceptions, distrust, religious beliefs, lack of awareness, and limited knowledge about vaccine-preventable diseases (VPDs), as well as the role of routine immunization (RI) in preventing them. Additionally, the complexity of immunization schedules and challenging service arrangements, which involve multiple appointments at different ages, can contribute to vaccine hesitancy and cause caregivers to default from following the routine immunization schedule [4–9].

Based on the 2016 Nigeria Multiple Indicator Cluster Survey (MICS), it was found that 42% of caregivers reported a lack of awareness as the reason for their children not receiving full immunization. Additionally, 11% expressed a lack of faith or trust in vaccinations [10].

Efforts to enhance vaccination outcomes are often classified into two main categories: interventions aimed at improving health service delivery and supply, and interventions targeting the demand for vaccines. Health service delivery and supply interventions typically involve enhancing human resources training and supervision, optimizing logistics, ensuring proper cold chain maintenance, and improving vaccine storage. On the other hand, interventions focusing on vaccine demand commonly include incentivizing vaccination or utilizing knowledge translation and education (KTE) strategies to encourage and maintain vaccine uptake [11]. The 2016 report on Global Routine Immunization Strategies and Practices (GRISP) highlights "community involvement" as one of the nine essential investments to achieve enhanced immunization outcomes [12]. Community involvement, in this context, refers to fostering a collaborative relationship between communities and immunization programs. This approach aims to ensure uniformly high coverage through the promotion of both high demand for vaccination and the delivery of quality services” [12]. It therefore becomes imperative to try or develop creative ways to involve communities for improved immunization coverage.

Community theater has proven to be an effective tool for promoting behavioral change communication [13, 14]. Community theater incorporates various elements such as acting, singing, dancing, and spoken poetry, which are performed in local settings like town halls and religious venues. When utilized effectively, it has the potential to enhance understanding and awareness of health issues, shape beliefs and attitudes that impact behaviors and social norms, encourage action, demonstrate recommended practices, promote the utilization of services, address misconceptions, and foster community support for recommended practices [15, 16]. Community theater can serve as a powerful method to facilitate positive changes in health knowledge, behavior, and associated social norms. It effectively promotes information sharing and encourages community dialogue [17–19]. Theater has the potential to enhance the effectiveness of health messages by adding a captivating and engaging element. It offers a compelling and relatable approach to explore various health issues. A well-executed theater performance has the power to not only shift people's perspectives but also influence their actions [20]. Theater actively engages the audience, capturing their attention and immersing them in a meaningful experience. It creatively delves into shared beliefs, diverse perspectives, and facilitates open discussions on sensitive topics [20, 21]. Theater provides a captivating and thrilling means for audiences to receive essential messages, transcending literacy barriers. It has the ability to engage individuals on both emotional and intellectual levels. The emotional response evoked plays a vital role in shaping attitudes and behaviors, but it is crucial to combine it with clear messages that encourage specific actions. Moreover, theater performances offer the advantage of repeated exposure to key messages, amplifying their impact, especially when delivered through multiple channels.

Utilizing human-centered design (HCD) is an effective approach to delivering theater performances that foster community engagement and ownership. HCD entails a creative and inclusive design process that places the end users at the forefront, ensuring their needs and perspectives are central to the development of products and services [22]. While initially developed and applied in industrial production, the utilization of human-centered design (HCD) has extended to health care settings, resulting in enhanced service delivery, expanded coverage, and improved program outcomes [23–29]. In the field of population health, the strategy entails collaborating with all stakeholders, particularly community members who are the intended beneficiaries of the promoted service, throughout the entire process, from conceptualization to the final development and presentation of the health intervention. The end users actively participate in all four phases of HCD—discover, define, develop, and deliver. This involvement is facilitated through various methods, including user observation, interviews, stakeholder mapping, brainstorm sessions, co-creation, and interaction prototyping [22].

The aim of this study was to educate caregivers on vaccines and vaccine-preventable diseases and empower them to seek immunization services as a right by engaging them through a human-centered design of trust building using community theater as a tool in Bayelsa and Rivers States in the Niger Delta region of Nigeria.

## Methods

### Study design

The community theater project was implemented as a quasi-experimental intervention—a before and after study—to ascertain the effect of community theater on immunization uptake. Our idea was built to test the hypothesis that caregivers will demand immunization services as a right if they are engaged through a human-centered process of trust building, education and social support. The intervention – community theater for immunization (CT4I)—aimed to empower caregivers to seek and fully utilize vaccination services by engaging them and their communities on VPDs and immunization using community theater that showcases real stories while taking cognizance of the social determinants of health that may influence their behavior.

### Our Human-Centered Design (HCD)

Our idea was birthed after a chance engagement with “Mama Koko,” a 36-year-old rural fisherwoman, who lived in the heart of the Niger Delta region of Nigeria and who had just lost her 3-week-old baby. During the engagement, Mama Koko talked about her pain and her regrets, watching her baby suffer convulsions and eventually die from neonatal tetanus, a disease that vaccination could have prevented. Mama Koko explained that her greatest regret was not knowing about tetanus and the vaccination that should have prevented it. HCD works with women like Mama Koko to co-create content that educates and amplifies positive stories that can increase demand for vaccination.

The HCD approach relied heavily on the collection and use of feedback for iterations of the initial model. An interesting lesson was drawn from the high attendance at plays by children aged 5—10 years, which resulted in deliberate efforts to modify the design to tap into the opportunities presented by the attendance of children. Messaging to support them as an audience that would engage their parents to vaccinate younger siblings, as well as for them to function as enthusiasts that would pave the way for increased acceptance of new vaccines in the region such as HPV when introduced and as champions that would safeguard their future and that of the next generation became a deliberate part of the storyboards.

As part of our human-centered design approach, there was a need to apply communication design through strategic engagement with the audience to design how vaccination messages were delivered during performances, in a consistent and engaging way. This resulted in changes from how the delivery of vaccination messages was originally structured – as dialogue—and expanded to include backdrops during performances and placards held up by children in specific scenes during the performances. There was also a need to make changes by applying experience design, focusing on the level of engagement and satisfaction that viewers derived from the performances while it addressed their needs and context. This was a key driver of testing the assumptions on repeat attendance, even though it was not a part of the original solution. What was arguably the biggest change to the solution was the decision to apply a lot more product design by researching, ideating, conceptualizing and building theater performances that better fit within the lives of caregivers and communities. This led to changes in locations where theater performances occurred, with a significantly higher leaning for religious houses and meetings of women groups, complemented by performances at routine community dialogue meetings.

Finally, there was an application of service design in the planning and organizing of people, infrastructure, communications, and material components of vaccination outreaches that were conducted at the theater session in order to improve the value, convenience, and interaction between health workers and caregivers. This was not part of the original solution but was the first change made to the solution after initial rounds of user research.

### Settings and study location

The intervention was implemented in two states in the Niger Delta region of Nigeria—Bayelsa and Rivers States. The Niger Delta in the South-South geopolitical zone of Nigeria is home to a number of under-immunized and zero-dose children, a situation that is attributable to the preponderance of hard-to-reach communities and security-compromised locales in the region.

Bayelsa state is located in the core of Niger Delta in Southern Nigeria with its capital as Yenagoa. It has a total area of 10, 773 km^2^ and a population density of 270.2 inhabitants per square kilometer. The estimated total population of the state for 2019 was 2,541,683 and the population of women of childbearing age (WCBA) was 559,170. The state comprises eight Local Government Areas (LGAs) and about 70% of the LGA are only accessible through waterways [30]. Rivers state is also in the Niger Delta region of Nigeria with its capital as Port Harcourt. The total area of the state is 11,077 km^2^ (4,277 Sq. mi) with a population density is 635.89 inhabitants per square kilometer. Its 2019 total population was 8,53,416 and women of childbearing age (WCBA) population was 1,947,752. The state is subdivided into 23 LGAs [31].

The intervention was intended for the lowest performing LGA in immunization coverage in each of the two states and we also planned to test the solution in one metropolitan LGA in each state. However, with funding constraints we could only implement in one metropolitan LGA. The study was therefore conducted in two LGAs in Bayelsa State – Ekeremor (lowest performing LGA) and Yenagoa (metropolitan LGA) and in Ahoada West LGA (lowest performing LGA) in Rivers State.

### Eligibility criteria for participants

#### Study population

The study involved all women of childbearing age (15–49 years) in the selected communities who were pregnant or had children 0–24 months of age and had lived in the community for at least six months prior to the household survey and the intervention which included participation as community cast and community champions. The audience for the performances was left open to all community members.

#### Sample size estimation

Within each LGA, we selected six priority communities based on a set of criteria that enabled us to test the effectiveness of our solution in rural vs. urban communities, in communities with poor vs. average performance on vaccination uptake, and in high-risk communities (hard to reach, security compromised, or frequently affected by floods) which typically contribute quite significantly to under- and un-immunisation in the region. A total of 18 communities were therefore included in the study. The Lot Quality Assurance Sampling (LQAS)methodology [32] was used to select 10 WCBA from 10 eligible households from each community using the probability proportional to size (PPS) for the pre- and post-implementation surveys and this gave a total sample size of 180.

#### Data collection

A mixed-method approach was used for data collection. This consists of quantitative data from household surveys, pre- and post-training tests and experiential feedback, and qualitative data from focus group discussion (FGD). Secondary data was also collected from the district health management information system (DHIS2). FGDs were conducted before the intervention and findings were used to develop storyboards and to design the survey instrument. Pre- and post-training tests were administered before and after training of community members and experiential feedback was collected after each performance. The household survey was conducted pre-intervention, midline (six months into the intervention) and endline (six months post-intervention).

#### Data collection tools

##### FGD

An FGD guide was used to facilitate the FGDs. The guide included questions about caregivers’ knowledge about vaccines and vaccine-preventable diseases, their knowledge of the child’s immunization schedule, their experience with accessing vaccination services before, during and after the pandemic, any barriers to accessing immunization services and how they think it can be better.

##### Pre- and post-test

A test script consisting of eight questions was used to test the potential routine immunization champion’s knowledge of vaccines and vaccine-preventable diseases before and after training. Questions were asked about immunization schedule, routes for vaccine administration, and possible adverse events that follow immunization.

##### Experiential feedback

A questionnaire was administered using the CoroSurvey app to collect user feedback on the plays. The questionnaire assessed the participants’ satisfaction with the plays, their willingness to pay to watch the play, how likely they are to recommend the play to their contacts, and how they feel about vaccines after watching the plays.

##### Surveys

A structured close-ended questionnaire was used to collect the survey data. The questionnaire included questions on the immunization status of the eligible child in the household, availability of child health cards and reasons for child’s incomplete vaccination if they were not fully vaccinated as well as caregivers’ source of information and trusted sources of information for immunization. The primary data source for the child’s immunization status was the child’s health card which was usually requested from the caregiver. The immunization status was assessed by checking the child’s health card which provides information on the child’s age as well as the antigens that the child has received and the date of the next visit. In instances where the child’s health card was not seen, the immunization status was assessed based on the caregiver’s account of the child’s immunization history. The conclusion was usually made after probing further into the response given by the caregiver such as the age of the child, how many visits and the injection sites or route of administration of the antigen during the last visit. This is to reduce inaccuracies to the barest minimum. The immunization status was classified as fully immunized, partially immunized or unimmunized. Fully immunized children are children who have received all the recommended antigens for their age, partially immunized children are children who missed out on one or more antigens for their age while un-immunized children are children who are eligible but have not received any dose of the recommended antigens.

#### Pre-intervention activities

We engaged with government stakeholders of the two states and the three LGAs to introduce the project to them. Initial engagements included key informant interviews with decision-makers and mid-level managers of the immunization and primary health care programs in these locales. The results of these interviews complemented desk reviews of existing national and state immunization program reports, helped to provide local context of the immunization program and highlighted both the demand- and supply-side challenges and current approaches to solving the problems.

Within the selected communities, there was an initial advocacy engagement with community leaders. The initial advocacy engagements provided a platform to secure the buy-in of the community and religious leaders and the buy-in of the community members at large. This ensured that our teams were accepted by intervention communities, and this became important following the outbreak of COVID-19 in Nigeria and the resulting hostilities from many communities in the region. The established relationships and the buy-in of the communities and their leaders secured through the advocacy engagements were key to allowing the communities open their doors and continuing to engage with us. It also provided the platform for target users in the communities to co-create with us on how to train for and perform plays safely in the context of COVID-19.

In all selected communities, a pre-intervention survey was conducted to obtain some quantitative data on immunization of children before intervention in those communities. The pre-intervention survey followed the LQAS methodology [32] where 10 eligible households were surveyed in each community. Eligible households here refer to households that have children between 0–24 months. The pre-intervention surveys were followed by a focus-group discussion (FGD) with women of childbearing age with at least one child between 0-24 months. The selection of participants for the FGD was guided by the health care workers and community leaders in the community. The FGD was designed to maximize demographic and functional diversity of the participants on the uptake of vaccination services. The FGD provided qualitative and experiential insights on the knowledge and perceptions of vaccines and vaccinations and on the unique barriers to accessing immunization services in each community.

#### The intervention

The intervention in the two states lasted for a period of 18 months, from November 2019 to May 2021. The key essence of the intervention was to apply creative stories and anecdotes that harness the powerful role emotions play in driving decision-making toward sustained positive behavioral change. The project set out to explore the potential of using storytelling and emotion in increasing demand for vaccination services and emphasizing how emotions can be harnessed to create long-lasting positive behavior change. The findings from the focus group discussions served as the basis on which the storylines for the Community Theatre for Immunisation (CT4I) presentations were built. Though several important themes emerged, the plays focused mainly on the theme (awareness) which we believe is most amenable to CT4I as an intervention.

The storyboards addressing barriers to immunization uptake in the communities were developed for the theater performances. A hero’s journey was co-created and clearly mapped out for each performance with two distinct hero’s journeys – a caregiver’s journey and a health worker’s journey. Participants from the FGD volunteered to audition as part of the community cast. The selected caregivers were given the chance to further share their experiences about the incompletion of immunization for their children. The selection of key actors was done with recommendations from the caregivers. The community cast were then trained on vaccine-preventable diseases, vaccination services and on key vaccination messages. They were also trained on theater performance and rehearsed using ad-lib dialogue in line with the storyboard and scenes that mapped the hero’s journey. Following this, the creative team formed tales that projected the anxieties of the caregivers while delivering pertinent answers to their concerns. We introduced their traditional songs, dances and drumbeats and allowed the performers to act in their local languages to remove communication barrier. The cast underwent a mini audition before performance to gauge their vocal and impersonation skills. This was followed by rehearsals where ideas and suggestions from the community cast were incorporated into the art piece to achieve the desired outcomes.

The theater performances targeted community dialogue meetings and religious gatherings (after Sunday service), as part of a design strategy to ensure that performances fit into the established routines of the communities*.* The theater performances included scenes that worked to make people feel *awe*, the overwhelming positive feeling of being diminished in the presence of something greater than the self—focusing on the role of vaccination in attaining health for the entire community. This was tied into theoretical projections that when we feel awe, we are more likely to be altruistic, self-reflective, open-minded and generous [33]. The performances included humor, which captures the attention of an audience to drive belief and behavior change toward an issue. This is because comedy is relatable, uses positive emotions and transports the viewer into the story [34]. The theater performances deliberately sought to demonstrate *pride*, which has been found to be effective in motivating people to engage in altruistic behavior to improve their image of self in the eyes of others, and to feel better about themselves [35]. The performances also told stories that tapped into universal values and emotions, like *love* for children and wanting to protect one’s family. These two emotions (pride and love) were amplified by telling stories of positive deviance in the utilization of vaccination services. An additional part of the performance was the discussion session and interpersonal communication facilitated by health workers to answer further questions from the caregivers and provide clarifications for the messages passed during the theater performances. At the end of each performance, experiential feedback was collected on the user’s experience during the play using a CoroSurvey App specifically developed for the intervention. The caregivers who served as community cast were charged to serve as community champions for routine immunization and to lend their voices to educate other caregivers and share accurate information on routine immunization in their communities.

#### Post-intervention

Mid- and post- intervention questionnaire surveys were conducted in the intervention communities. The midline survey was conducted six months into the intervention period and the endline survey six months afterward. The post-intervention phase also included a dissemination meeting where study findings were presented to midlevel immunization managers and state decision-makers.

#### Data analysis

Quantitative data were entered and analyzed using Microsoft Excel. Frequencies and proportions were generated for categorical variable. The audio-taped FGDs were transcribed and thematic analysis was done manually generating themes from the transcripts. Four broad themes emerged and these formed the themes for the storyboard of the community theater.

## Results

### Initial engagement meetings

In the 18 communities involved in the three LGAs in the two states, 32 planning meetings were held and 56 midline immunization managers and 59 traditional and religious leaders were engaged.

### Focus group discussion

A total of 18 FGDs were conducted—one per community with an average of 12 participants at each FGD. A total of 216 women of childbearing age participated in the FGDs. Table 1 shows the summary of findings from the FGDs in the 18 communities. The barriers to vaccination identified from the FGDs were categorized into four broad themes – Awareness, trust, motivation, and action.

**Table 1:** Result showing thematic analysis of FGDs

Themes	Issues Identified
**Awareness**	Misconceptions
	Knowledge gap on VPDs and immunization schedule
	Inadequate knowledge on the management of adverse event following immunization (AEFI)
**Trust**	Quality of services
	Health worker unavailability
	Over-dependence on traditional medicine
	Doubt about health worker competencies
	Poor attitude of health workers
**Motivation**	History of previous AEFI
	Social influence (refusal from husband and relatives)
	Stock out of vaccines causing long waiting hours at the facilities
	Poor state of health facilities
	Conflicting activities
**Action**	Service charge
	Distance from health facility
	Poor reminders

### Awareness

The common issues highlighted under the theme of awareness were misconceptions about vaccines. Some caregivers said that immunization caused the death of their children, some said it resulted in paralysis of their children while some others stated that immunization was not safe for them. We also had caregivers who lacked accurate knowledge on the diseases that vaccines can prevent; some said that vaccines could prevent typhoid fever, malaria and convulsion. Other caregivers did not know the number of visits required to complete their children’s vaccination or what to do in case of adverse event following immunization (AEFI).

*“I don’t know anything about immunization”-Caregiver, Yenagoa FGD.*

*“Immunization is the treatment given to children at the health center when they are sick” – Caregiver, Yenagoa FGD*

To also understand how much the caregivers know about immunizations, they were also asked about the immunization schedule and how many visits they need for their children to be fully immunized.

*"I don’t know how many times I need to go to but I have gone like three times” – Caregiver Ahoada-West FGD*

### Trust

Some caregivers reported a lack of trust in the health system while doubt in the competencies of health workers was mentioned by others as the reason why they did not utilize vaccination services. They also noted that sometimes the health centers are locked, they would’t know whether they are still functional or not. Other reported lack of trust in the vaccines and the general safety of needles used for the injection.

*I don’t trust the nurses in my health center- Caregiver, Ahoada-West FGD*

*“My husband discouraged me. He does not believe in immunization”- Caregiver, Ekeremor FGD*

### Motivation

On the theme of motivation, caregivers reported previous AEFI such as high fever, swelling, excessive cry and restlessness; social influence like husband or mother in-law's refusal and pastor discouraging vaccination, as well as long waiting hours at the health facilities as deterrent or demotivators for starting or completing vaccination.

*“My people do not bring their children to the hospital. They are scared that after vaccinating their children, when they get home, the child will die, they prefer to use native medicine to cure their children because the children usually suffer when they get immunized” – Caregiver Yenagoa FGD*

The adverse events following immunization were predominantly the major reasons that prevented the caregivers from utilizing available vaccination services.

*“Why I don’t immunize is because when I immunized my child, it affected my child, the hand was swollen, and pus was extracted from it, and my child could not raise his hand and he was always crying and disturbing”.-Caregiver, Ahoada FGD*

### Action

The distance caregivers had to travel to get to the nearest health facility and inability to remember child’s next visit to the immunization clinic were mentioned by some caregivers as reasons why they defaulted and could not complete the immunization schedule for their children. Another reason given for incomplete vaccination was lack of money to pay service charge requested in some places.

*“When you carry your child to the health center, you have to wait for so long, when they attend to you, the injection or the tablet will not be effective”. Caregiver, Yenagoa FGD*

### Storyboards

Twenty-nine storyboards were developed from the themes identified from the FGDs using the hero’s journey of two heroes, the caregiver and the health worker. The stories were co-created with the community members and the community cast.

### Theater training and performances

A total of 217 community members were trained on routine immunization and theater performances across the 18 communities. These community members were caregivers (mothers) who had children 0–24 months. Change in their knowledge was measured using a pre- and post-training test administered before and after each training. Overall, 72% of the trained community members had a knowledge increase. Table 2 shows the number of community members engaged and the number of caregivers trained on routine immunization and theater performance in each LGA and community. Ahoada-West LGA and Okarki community, both in Rivers State had the largest number of community members engaged and caregivers trained. A total of 29 performances were conducted in the 18 communities with 2,258 caregivers (women) in attendance out of which 205 (9.1%) were identified as pregnant (Table 3).

**Table 2:** Number of community members engaged, and caregivers trained in each community

LGAs	Communities	Number of community members engaged	Number of caregivers trained
**Ekeremor LGA**	Aleibiri	195	17
	Ayamasa	198	21
	Tamogbene	79	6
	Toru-Ndoro	117	15
	Ekeremor	127	16
	Peretorugbene	121	10
	**Total**	**837**	**85**
**Yenagoa LGA**	Azikoro	56	8
	Kpansia	118	5
	Zarama	201	15
	Kalaba	48	8
	Yenaka	86	12
	Yenegwe	58	5
	**Total**	**567**	**53**
**Ahoada-West LGA**	Okogbe	152	7
	Odawu	120	16
	Odiokwu	139	10
	Upatabo	79	13
	Mbiama	91	5
	Okarki	444	28
	Total	1, 025	79
	**Grand Total**	**2,429**	**217**

**Table 3:** Characteristics of play participants and experiential feedback from the play

LGA	Ahoada West (n, %) *n* = 750	Ekeremor (n, %) *n* = 908	Yenagoa (n, %) *n* = 600	Total (n, %) *N* = 2258
**Pregnancy Status**				
Pregnant	45 (6.0)	136 (15.0)	24 (4.0)	205 (9.1)
Not pregnant	705 (94.0)	772 (85.0)	576 (96.0)	2053 (90.9)
**General feeling about the play**				
Satisfied	653 (87.1)	726 (80)	522 (87.0)	1901(84.2)
Unsatisfied	97 (12.9)	82 (9.0)	78 (13.0)	257 (11.4)
Neutral	0	100 (11.0)	0	100 (4.4)
**Feeling about the length of the play**				
Adequate	533 (71.1)	572 (63.0)	468 (78.0)	1573 (69.7)
Too long	217 (28.9)	336 (37.0)	132 (22.0)	685 (30.3)
**Willingness to pay 100 naira for the show**				
Yes	585 (78.0)	617 (68.0)	348 (58.0)	1550 (68.6)
No	165 (22.0)	291 (32.0)	252 (42.0)	708 (29.1)
**Preference for the play to be done at night**				
Yes	128 (17.1)	200 (22.0)	48 (8.0)	376 (16.7)
No	622 (82.9)	708 (78.0)	552 (92.0)	1882 (83.3)
**Feeling of vaccine acceptance**				
Yes	622 (82.9)	717 (79.0)	522 (87.0)	1861 (82.4)
No	128 (17.1)	191 (21.0)	78 (13.0)	397 (17.6)

### Feedback/Outcome of the theater sessions

Experiential feedback collected at the end of the performances shows that 84.2% (1901/2258) of the participants were satisfied with the performances, 69.7% (1573/2258) reported the length of play to be adequate and about two-third (68.6%,1550/2258) of participants reported willingness to pay the equivalence of a bottle of soda to watch the play, while 16.7% (376/2258) of all attendees wanted future performances to happen at night. The majority (81%) of the engaged community members felt more accepting of vaccine for their children following the community theater intervention. At the plays, 391 persons were vaccinated among whom were 270 children. Out of the 270 children, 62 (23%) were zero-dose children—receiving vaccines for the first time during the play.

### Survey findings

**Fig. 1:**
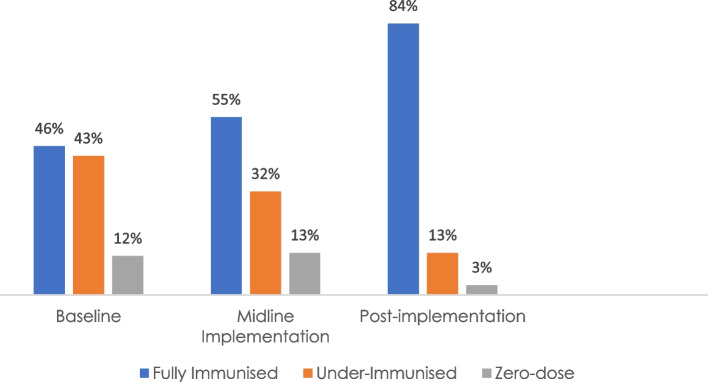
Changes in immunization status of children in the intervention communities

Table 4 shows the proportion of caregivers who reported lack of information, lack of trust or lack of motivation as reasons for their child’s incomplete vaccination at baseline, midline and endline surveys across the three LGAs. The table shows a decrease in the proportion reporting lack of information and motivation from pre-intervention to midline but an increase in the proportion from midline to endline. No participant reported lack of trust at baseline and at endline surveys but five caregivers (8.3%) each in Ahoada and Ekeremor LGAs mentioned it at the midline survey. Figure 1 illustrates the changes in children’s immunization status in the communities before and after the intervention. There is an appreciable increase in the number of fully immunized children from 46% at baseline to 55% at midline and to 84% post-intervention. In 65%, 70% and 75% of the households, child health cards were seen to assess the child’s immunization status for baseline, midline and endline surveys respectively. Where the child’s health card was not seen, the caregivers’ account of the child’s immunization history was used to assess the child’s immunization status.

**Table 4:** Reasons for child’s incomplete vaccination

**Reason**	**Ahoada** ***N* = 60 (**%**)**	**Ekeremor** ***N* = 60 (**%**)**	**Yenagoa** ***N* = 60 (**%**)**	**Total** ***N* = 180 (**%**)**
**Lack of information**				
Pre-implementation survey	23 (38.3)	34 (56.7)	17 (28.3)	74 (41.1)
Midline implementation survey	0	25 (41.7.)	0	25 (13.9)
Endline implementation	10 (16.7)	15 (25.0)	0	25 (13.9)
**Lack of trust**				
Pre-implementation survey	0	0	0	0
Midline implementation survey	5 (8.3)	5 (8.3)	0	10 (5.6)
Endline implementation	0	0	0	0
**Lack of motivation**				
Pre-implementation survey	24 (40)	17 (28)	33 (55)	74 (41.1)
Midline implementation survey	11 (18)	0	25 (42)	36 (20.0)
Endline implementation	20 (33)	25 (42)	20 (33)	65 (36.1)

## Discussion

A lot of resources have been invested to improve immunization coverage among children in Nigeria but recent national surveys still show unacceptably low coverages in many states of the country including Bayelsa and Rivers States. Our study was designed to test an uncommon approach that has the potential to improve immunization coverage by reducing vaccine hesitancy among caregivers. In the target localities for this intervention in Bayelsa and Rivers States in the Niger Delta region of Nigeria*,* our creative team worked with the leadership of the health system, the community leaders, the health workers and community members to create and perform theater. Rural women and community members acted out the plays that they have co-created, to increase awareness, rebuild trust and motivate pregnant women and caregivers to actively demand vaccination services**.** The meetings, focus group discussions and the baseline survey revealed the different barriers to the utilization of available immunization services. These engagements with the people help to “discover” the problem and to define it more explicitly in the spirit of human-centered design thus giving opportunity to iterate lessons that were used in developing and improving the intervention [22].

Similar to findings from previous studies [11, 36], misconceptions, lack of awareness and poor knowledge of vaccine preventable diseases and the immunization schedule came out as important factors that needed to be addressed in the study communities and were targeted in the intervention. Frequent disruptions in the operation of immunization clinics, vaccine stock-out and poor attitude of health workers seemed to affect the trust caregivers in the communities had in the immunization process. As reported previously, distrust in the health system could negatively affect service utilization [11]. Adverse event following immunization (AEFI) was a factor discovered in the area as contributing to low immunization uptake. Occasionally, children experience AEFI when vaccinated but when this is moderate to severe and the caregiver is not adequately informed about it, hesitancy or failure to complete the schedule is inevitable. The study was conducted in rural areas where tradition, beliefs (cultural and religious) and significant others play a role in the health seeking behavior of the people. It is no surprise then that husbands, mothers-in-law and religious leaders were mentioned as influencing vaccination of children. Poor geographical and financial access have been reported as barriers to immunization. There was no functional health facility in some of the intervention communities and presumably, they were not adequately covered by immunization outreach that is expected to serve such remote areas. Even though immunization is “free,” lack of money for round trips to the nearest vaccination post and for sundry charges collected at some facilities may discourage caregivers from taking their children for immunization.

All of the findings above were considered in the development of the intervention – theater performance. The caregivers who formed the community cast were trained on routine immunization and theater performance and the majority of them showed a knowledge increase in comparison to a study that used a similar approach [13]. The theater performances were acted by these caregivers in community townhall, religious houses as well as market squares to educate the communities on vaccines and vaccine preventable diseases. Beyond the play, the actors, now regarded as community champions for routine immunization, continued to create more awareness, address misconceptions and promote accurate information on routine immunization in their communities.

Immediate experiential feedback after the plays showed acceptability of the intervention given the proportion of participants that were satisfied with it and that were more accepting of vaccination. In fact, some children received their first ever vaccination at the theater. Sustaining an intervention like this may be a challenge without some incentive for the actors that had to sacrifice their time for training, rehearsals and the actual plays. Since about a third of play participants were willing to pay some amount of money to watch the play, money generated from there could be used to incentivize play actors and to aid organization of future performances. HCD entails iteration and continued user testing of the play prototype already developed and this is essential to address imperfections that probably left some participants unsatisfied with the play [22].

The intervention apparently led to a decline in the proportion of women reporting lack of information as a reason for their child’s incomplete vaccination. Lack of motivation also diminished, albeit inconsistently, across the survey periods. Contrary to the findings of the FGDs, where trust issue was identified as a theme, the survey did not show lack of trust as a major hindrance to vaccination. FGD allows people to freely express themselves and bare their minds in a way that is impossible with a questionnaire survey; it is possible that the FGD participants used the opportunity to discuss lack of trust in the health system as a deeper issue of concern to them. Moreover, the study fell within the COVID-I9 period which was marked by conspiracy theories, some of which indicted the government and led to a distrust of many citizens in public institutions and their activities [36, 37]. This general distrust could be what was the women expressed at the FGDs.

Overall, there was an increase in the proportion of fully immunized children and a decline in the proportion of under-immunized and zero-dose children. The dissemination meetings enabled the state immunization leadership to see the depth of the reasons behind vaccine hesitancy among caregivers in the states and the extent to which the intervention had helped to address the barriers to vaccination demand in the intervention communities.

### Limitations

Our study has some limitations which should be considered while reading and making sense of our findings. First, the states and LGAs (districts) of intervention were purposively selected and were not representative of the states in the Niger Delta or the LGAs in the two selected states. This limits the generalizability of our findings. However, the LGAs are somewhat homogenous in the urban/rural strata and the intervention could work with some modifications to give similar results in non-intervention LGAs. The purposive selection of intervention LGAs should therefore not deter scalability of the intervention. Second, social desirability bias could not be ruled out as influencing the response that greeted the play leading to positive experiential feedback and a number of persons being vaccinated at the play. The performances used evoked a lot of positive emotions that could have prompted the response. It is not certain whether the emotions and positive response could be sustained, but with the co-creation process and the expected continued activity of the community champions, there is reason to hope for the maintenance of the response and the improved immunization uptake. Third, the intervention focused mainly on caregivers which could easily be reached through community theater. Addressing supply-side factors discovered at the pre-intervention phase could potentially synergistically improve vaccine uptake in the intervention communities but this is outside the scope of the study. Fourth, apart from the engagement meetings that involved some males, the community cast were all women and the performances were exclusively attended by females. This detracts from the additional effect active male involvement in the co-creation process, acting and attendance at the play could have given. Lastly, some of the children’s immunization status was assessed on caregivers’ account of the child’s immunization history because the child’s health card could not be found. We recognize that this could be subject to recall bias which may also affect the changes in immunization status recorded.

## Conclusion

The Community Theater for Immunization intervention was built to test the hypothesis that caregivers will demand immunization services as a right, if they are engaged through a human-centered process of trust building, education and social support**.** Results from the intervention communities demonstrate an increase in knowledge among caregivers who were trained and acted in the play and a decline in the proportion of caregivers who reported either lack of information or lack of motivation as a reason for their child’s incomplete vaccination. Results also shows important experiential results in deploying theater through a human-centered process, with reported satisfaction and increased acceptance of vaccination by attendees at the play and the utilization of available vaccination services provided at the theater performances.

Communities were engaged to take the lead in identifying some of the demand-side challenges and barriers to demanding immunization, and these topics formed the foundational themes around which drama episodes were developed. Ultimately, the intervention leveraged the power of story-telling and the potential role of design thinking to address the global challenge of increasing demand for vaccination.

### Supplementary Information

**Additional file 1: Appendix 1.** A sample play script.

## Data Availability

The datasets used and/or analyzed during the current study are available from the corresponding author on reasonable request.

## Abbreviations

WHO: World Health Organization
NDR: Niger Delta Region
CT4I: Community Theater for Immunization
RI: Routine Immunization
SDGs: Sustainable Development Goals
LMICs: Lower-middle-income countries
VPDs: Vaccine-preventable Diseases
MICS: Multiple Indicator and Cluster Survey
KTE: Knowledge Translation and Education
GRISP: Global Routine Immunization strategies and Practices
HCD: Human-centered Design
LGAs: Local Government Areas
LQAS: Lot Quality Assurance Survey
PPS: Probability proportionate to Size
FGD: Focus Group Discussion
DHIS2: District Health Information System 2
COVID-19: Corona Virus Disease 2019
AEFI: Adverse Events Following Immunization
