# Supplementary material for: Using community theater to improve demand for vaccination services in the Niger Delta Region of Nigeria
Source: BMC Proc. 2023 Jul 3;17(Suppl 7):6. doi: 10.1186/s12919-023-00263-0 (PMC10316554; doi:10.1186/s12919-023-00263-0)
Supplement: Supplementary file 1 — Additional file 1: Appendix 1. A sample play script. [file 12919_2023_263_MOESM1_ESM.docx]

**Using Community Theater to improve demand for Vaccination Services in the Niger Delta Region of Nigeria**

**Appendix 1: A sample play script**

ZARAMA PLAY

(A true-life event)

SC. 1

Mummy Bliss is seen at the corridor of her home, she is sitting on a mat, carrying a tray of melon seeds on her laps, and she is seen peeling and bland blowing out the chaff. Mummy Bliss hears a slight sobs coming from the direction of her room

MUMMY BLISS: E! Be like say this girl don wake, Abeg make she stay there rest, abi mama wey born pikin no go rest

Mummy Bliss continues to blow off the chaff, her baby’s cry intensifies and this time it is louder and sharper, out of fear she drops the tray of melon seeds on the ground, and dashes into the her room. She comes out carrying her baby on her shoulder, the baby is still crying

MUMMY BLISS: Shussssssh... My pikin na who beat you? You con fear me

The baby continues to cry, A man wearing an immunisation apron, he is holding a mega phone, he is identified as an house to house Mobiliser, he walks towards her

MUMMY BLISS: Oga! No vex oh! Who you dey find?

MOBILISER: Na you I dey find oh!

MUMMY BLISS: How you go find person wey no sabi you? I no know you

MOBILISER: Okay madam! I be! House to House Mobiliser for Zarama health centre

MUMMY BLISS: okay, wetin you con find here

MOBILISER: Madam Wetin dey do your pikin, since I enter, en just dey cry

MUMMY BLISS: Oga! That one no concern you, my own be say wetin carry you come?

MOBILISER: I dey go house to house dey invite women them wey get small pikin them, make them arrange themselves say tomorrow na immunisation for health centre

MUMMY BLISS: First! who tell you say small pikin dey here.... Second! who tell you say I sabi wetin be immunization

Mobiliser tries to answer, she cuts in the conversation

MUMMY BLISS: thank god say you see as my pikin dey cry, I no get your time, I if you no get anything to talk again, I been dey busy when you come meet me

MOBILISER: Madam see en, as I been dey come na your neighbor show me say you get small pikin for this house, and for immunisation I no no about that one

MUMMY BLISS: Oga! I no dey give my pikin something wey I no no, my mama wey born me, no give me, so why I go come carry my pikin go una health con give am the thing you dey talk, oga as you see me so, I no be health person

MOBILISER: Ah ah! Madam, as you know know about this immunization, na for health centre them go tell you wetin e be, madam this immunization dey help protect children from sickness

MUMMY BLISS: I tell you say y pikin dey sick, oga abeg just comot, I fact make I come leave you here

She picks up the tray, and her mat, and exits the stage; the Mobiliser also leaves the stage

SC.2

Same Day, In the evening, a woman walks towards the stage carrying a small nylon bag, she calls out for Mummy Bliss

MOTHER INLAW: House oh! People dey this house at all

Mummy bliss from inside, Mummy bliss comes out

MUMMY BLISS: Mama welcome oh! So vex oh! I think say na person I been pusue before, How you dey?

MOTHER INLAW: hmmmm... I dey fine, my pikin dey house…

MUMMY BLISS: no oh, en never come back from shop, make we go inside

Mummy bliss starts crying again

MUMMY BLISS: OOOOOOOOh! I think say this girl don sleep, which kind wahala be this

MOTHER INLAW: wetin happen

MUMMY BLISS: Mama since morning this pikin don dey cry, body don hot finish eye don red join, mama make I go carry her come

MOTHER INLAW: Okay my pikin, bring chair come too, I wan stay outside make breeze blow me, make I carry my grand pikin

Mummy bliss goes in, carrying the bag of the mother inlaw, comes out with her baby wrapped at her back, she is sen carrying a plastic chair out, she places the chair, her mother inlaw sits

MOTHER INLAW: Thank you, give me my pikin make I carry jare

Mummy bliss, unwraps her baby and gives her mother inlaw her baby

MOTHER INLAW: Jesus Christ! The body too hot oh! try carry her go health centre oh, make them check her

MUMMY BLISS: Mama, no need for health centre, I dey plan go my mother side, na my husband I dey wait for am to come back

MOTHER INLAW: Your mother na Doctor!

MUMMY BLISS: Hahahaha... Mama, my mother no be doctor, but she dey do native medicine, mama you see this her native medicine her, na wetin she use born us, all of no sabi road to health, na why I tell one guy wey tell me say make I carry my pikin go health for immunisation, I tell am say I no fit give my pikin something we my mama no give me when I small

MUMMY BLISS: Mama you know wetin immunisation be?

MOTHER INLAW: My pikin, my mama wey born me no sabi immunisation na why she go give me, and na why I no give my children including your husband, but now I know about am, from my friend wey her pikin just born, me and her been dey when her pikin call say pikin dey sick, say she dey health centre, na so my friend say make I follow her go health, as I reach there, I see plenty women them, I con ask wetin dey happen there, them say na immumisation, say na something wey them dey give children to protect them from bad bad sickness, na so I tell my friend say make she tell her daughter to give their pikin after them treat the pikin wey sick

MUMMY BLISS: Na wa oh, but ma na the immunisation cure the sickness na?

MOTHER INLAW: No be am, them treat the pikin first before them con immunise am, and them immunize am because en temperature don come down

MUMMY BLISS: Okay oh! But I never go health before na native I believe so oh

MOTHER INLAW: you know wetin dey do this pikin wey you wan carry am go native

MUMMY BLISS: My mother go sabi oh!

MOTHER INLAW: My pikin time dey go oh, you see this your pikin I carry so, na my graund pikin and I want am to dey feel fine, you know joe, your husband elder brother

MUMMY BLISS: Yes!

MOTHER INLAW: Na so en last pikin nearly die, because of say them delay to go health centre, pikin dey sick, them dey treat pikin for house, them no even know wetin dey wrong sef, but when them carry the pikin go health, na so doctor see wetin dey do pikin, them con treat am pikin con dey alright

MUMMY BLISS: You know the kind sickness

MOTHER INLAW: I no fit talk am na oyigbo name, e too long

MUMMY BLISS: Them give the pikin the immunisation?

MOTHER INLAW: yes but no be that day, as I hear about am na so I tell am too, but I just dey wonder why I no tell you on time, but e never late, carry my grandchild go health make them check wetin dey do am, en body still dey hot, even if say the cry don reduce, you still need carry am go health now, from there them fit give am the immunisation

MUMMY BLISS: Okay but I need to tell my husband where I dey go

MOTHER INLAW: no worry I still dey here, I go just enter house wait am for parlour, just go change then go health

MUMMY BLISS: Okay make me enter house

Both mummy bliss and her mother inlaw goes into the house carrying the chair, while mummy bliss walks out wrapping the baby at her back, she leaves the stage

SC. 3

At the Health centre, a table and two chairs are seen on the stage, with mentholated spirit, cotton wool, syringes, few vitamins, child registers etc are seen on the table, mummy bliss walks in, the RI FP walks towards her, she orders her to sit, while she sits also

RI FP: Good after evening madam!

MUMMY BLISS: Good after noon sister

RI FP: Wetin I go do for you and your pikin...

MUMMY BLISS: sister my pikin body dey hot am, and she dey cry well well, eyes don red finish, sometimes she go stop to cry,en no go fit sleep sef, next thing she done start, my mother inlaw say make I carry am come here, say una know wetin to do

RI FP: E good as you carry her come, make I check her temperature (She checks the child using a thermometer) her temperature high oh, apart from these symptoms you talk so, madam you notice another thing for the pikin body

MUMMY BLISS: no oh!

RI FP: Okay na! we go do small test (she carries the RTD Kit) I go use this thing check am, make I collect small blood for en hand

MUMMY BLISS: I hope say en no go pain am

RI FP: E go pain but na small thing

MUMMY BLISS: Okay na

The RI FP collects the drop of blood from the child

RI FP: Madam this your pikin na how old en dey?

MUMMY BLISS: Na big girl oh!, she be 9 months

RI FP: correct girl, you dey give this your big girl immunisation so?

MUMMY BLISS: Sister you see eh, this immunisation my mama no give me when I small, and na why I go give my pikin

RI FP: But you dey hear about am na

MUMMY BLISS: I no dey hear about am, I no ever know wetin e be until today, one man con invite me for house to come health tomorrow for the immunisation but I tell a, say I no dey give my pikin something my mama no give me but my mother inlaw tell me say them tell her say this immunisation dey protect children from bad bad sickness, and the wey she talk am, she dey really serious about am

RI FP: you con belive am?

MUMMY BLISS: Yes na, she na mama, she tell me say she no take am, her son wey I marry no take am but her grand pikin wey I born so go take am, because she hear say na very good thing, me sef I come sef to know wetin dey do my pikin plus to give am the immunisation too

RI FP: I dey happy say your mother inlaw convince you to come here so, your pikin get malaria and this malaria na sickness wey mosquito dey give person, when e bite person

MUMMY BLISS: You mean am say malaria dey do small pikin too

RI FP: yes na, no be only adult dey get malaria, and e no good for small pikin like this to get am, because e dey too dangerous, madam you go try to dey cover any water wey no dey move, wey con go stay one play dey smell, cover am with sand, then make una as family, yourself, your husband and una pikin, make una dey sleep for mosquito net that way mosquito to go fit bite any of una

MUMMY BLISS: no wahala my sister I don hear wetin you talk so, but if my pikin take this immunisation e go protect am from malaria na?

RI FP: No oh! Malaria na sickness wey immunisation no dey protect, na sickness wey mosquito they cause, you wey no take immunisation dey get malaria talk more of pikin, immunisation get different diseases wey e dey protect, sickness like measles, like pneumonia, like polio, like tetanus, like whooping cough, them many, and pikin wey get this kind sickness if them no come health, the sickness fit kill them, some of them they blind pikin eye, some they break leg sef, this sickness na immunisation dey prevent children from am

MUMMY BLISS: Sister abeg give am, I no want make my pikin sick that kind sickness oh

RI FP: you go carry am come tomorrow, na tomorrow be the immunisation day, we go register am and that one na free, and madam as your pikin never take any immunisation before na nine shots we go give am oh

MUMMY BLISS: Wetin be nine shots?

RI FP: I mean immunistion we go give am nine shots, because en never take when e small, pikin suppose take immunisation from when them born am, then in six weeks time, then ten weeks, 14 weeks 6months, nine months, and last for 15months, but since your pikin na just nine months e day, we go give am the immunisation wey en on miss then when en reach 15 months en go come take another one wey be the last one

MUMMY BLISS: Hmmm na wa oh, them many oh, but as long say my pikin no go sick all this bad sickness

RI FP: Before you go, you go buy malaria drugs, make you for dey give am

MUMMY BLISS: Okay!

The RI FP gives her some drugs and directs her on how to take it, then mummy bliss collects the drugs, she pays, thanks the RI FP and leaves the stage

SC. 4

At the health centre the next day, the RI FP is seen standing in front of a table which is has the Giostlye on it, the data tools, safety box, syrings, cotton wool, immunisation card etc, women are seen sitting which includes mummy bliss

RI FP: Good morning my mothers, how una dey?

WOMEN: We dey fine oh!

RI FP: Before we start we go first sing make person lead us for song

A woman volunteers, the women sings

RI FP: Make other person pray for us make we for start, oh see my friend

She calls mummy bliss to pray who prays, then she begins the health talk, talks more on immunisation, the different VPDs, Talks about Malaria not being a VPD, educates women on how to prevent malaria from their babies

The RI FP, rounds up, she requests for their immunisation card, woman goes to give her

MUMMY BLISS: I no get oh, you say na today you go give me

RI FP: yes, oya come this side, na you come first na you I go attend to first, na first come first serve, she registers mummy bliss child in a card, then proceeds to vaccinate the child

MUMMY BLISS: This thing go pain oh!

RI FP: E go pain, after this immunisation buy paracetamol give am for the pains

*The RI FP gets the child immunised with the nine shots, she tells her to come in 15 months time, and she should always look at the card to when is the time, the RI FP immunizes other children, then she closes the session.*
